# Clinical and Molecular Characteristics of Childhood-Onset Stargardt Disease

**DOI:** 10.1016/j.ophtha.2014.08.012

**Published:** 2014-10-12

**Authors:** Kaoru Fujinami, Jana Zernant, Ravinder K. Chana, Genevieve A. Wright, Kazushige Tsunoda, Yoko Ozawa, Kazuo Tsubota, Anthony G. Robson, Graham E. Holder, Rando Allikmets, Michel Michaelides, Anthony T. Moore

**Affiliations:** 1 Laboratory of Visual Physiology, National Institute of Sensory Organs, National Hospital Organization, Tokyo Medical Center, Tokyo, Japan; 2 Department of Ophthalmology, Keio University, School of Medicine, Tokyo, Japan; 3 UCL Institute of Ophthalmology, London, UK; 4 Moorfields Eye Hospital, City Road, London, UK; 5 Department of Ophthalmology, Columbia University, New York, New York; 6 Department of Pathology and Cell Biology, Columbia University, New York, New York

## Abstract

**Purpose:**

To describe the clinical and molecular characteristics of patients with childhood-onset Stargardt disease (STGD).

**Design:**

Retrospective case series.

**Participants:**

Forty-two patients who were diagnosed with STGD in childhood at a single institution between January 2001 and January 2012.

**Methods:**

A detailed history and a comprehensive ophthalmic examination were undertaken, including color fundus photography, autofluorescence imaging, spectral-domain optical coherence tomography (SD-OCT), and pattern and full-field electroretinograms. The entire coding region and splice sites of *ABCA4* were screened using a next-generation, sequencing-based strategy. The molecular genetic findings of childhood-onset STGD patients were compared with those of adult-onset patients.

**Main Outcome Measures:**

Clinical, imaging, electrophysiologic, and molecular genetic findings.

**Results:**

The median ages of onset and the median age at baseline examination were 8.5 (range, 3–16) and 12.0 years (range, 7-16), respectively. The median baseline logarithm of the minimum angle of resolution visual acuity was 0.74. At baseline, 26 of 39 patients (67%) with available photographs had macular atrophy with macular/peripheral flecks; 11 (28%) had macular atrophy without flecks; 1 (2.5%) had numerous flecks without macular atrophy; and 1 (2.5%) had a normal fundus appearance. Flecks were not identified at baseline in 12 patients (31%). SD-OCT detected foveal outer retinal disruption in all 21 patients with available images. Electrophysiologic assessment demonstrated retinal dysfunction confined to the macula in 9 patients (36%), macular and generalized cone dysfunction in 1 subject (4%), and macular and generalized cone and rod dysfunction in 15 individuals (60%). At least 1 disease-causing *ABCA4* variant was identified in 38 patients (90%), including 13 novel variants; ≥2 variants were identified in 34 patients (81%). Patients with childhood-onset STGD more frequently harbored 2 deleterious variants (18% vs 5%) compared with patients with adult-onset STGD.

**Conclusions:**

Childhood-onset STGD is associated with severe visual loss, early morphologic changes, and often generalized retinal dysfunction, despite often having less severe fundus abnormalities on examination. One third of children do not have flecks at presentation. The relatively high proportion of deleterious *ABCA4* variants supports the hypothesis that earlier onset disease is often owing to more severe variants in *ABCA4* than those found in adult-onset disease.

Stargardt macular dystrophy (STGD) is the most common form of juvenile-onset macular degeneration; it is inherited as an autosomal-recessive trait and caused by mutations in the *ABCA4* gene.^1–3^ Most cases present with central visual loss in early teenage years and ophthalmoscopy classically reveals macular atrophy with yellowish-white flecks at the posterior pole at the level of the retinal pigment epithelium (RPE).^1^

A large number of studies have described wide phenotypic variability and variable severity in *ABCA4*-associated retinopathy. The various phenotypes encompass macular atrophy without flecks, bull's-eye maculopathy, fundus flavimaculatus (retinal flecks without macular atrophy), a foveal sparing phenotype, cone-rod dystrophy, and “retinitis pigmentosa.”^1–20^ There is also considerable allelic heterogeneity, with >700 variants in *ABCA4* having been reported to date. ^1,2,4–34^

Patients with childhood-onset STGD tend to develop early severe visual acuity (VA) loss, markedly compromised retinal function on electroretinography with generalized rod and cone system dysfunction, and rapid enlargement of RPE atrophy and progressive loss of retinal function.^5,10,13,35,36^ Patients with adult-onset disease are more likely to retain useful VA for longer and show milder retinal dysfunction at diagnosis.^7,11,13,15,35^ There have been no previous studies specifically describing the clinical findings in a large cohort of molecularly confirmed STGD patients presenting and examined in childhood; the majority of previous reports relate to clinical features of patients examined in adulthood, some of whom may have had childhood-onset disease.

The purpose of this study was to describe the detailed clinical and molecular genetic findings of a large cohort of patients from a single center with childhood-onset STGD examined before 17 years of age.

## Methods

### Patients

Forty-two patients diagnosed with STGD at <17 years of age, between January 2001 and January 2012, were ascertained from the pediatric inherited retinal disease clinics at Moorfields Eye Hospital. Two subjects have been described in a previous case report.^9^ Blood samples were collected and genomic DNA extracted from peripheral blood leukocytes after obtaining informed consent. The protocol of the study adhered to the provisions of the Declaration of Helsinki and was approved by the local Ethics Committee of Moorfields Eye Hospital.

### Clinical Evaluation and Electrophysiology

A detailed medical history was obtained and a full ophthalmologic examination performed. The age of onset was defined as either the age at which visual loss was first noted by the patient or, in the “asymptomatic” patients, when an abnormal retinal appearance was first detected. The duration of disease was calculated as the difference between age at onset and age at most recent examination in childhood. The follow-up data were obtained before the age of 17 years.

Clinical evaluation included best-corrected VA, dilated ophthalmoscopy, color fundus photography, fundus autofluorescence imaging (AF), spectral-domain optical coherence tomography (SD-OCT), and electrophysiologic assessment. Best-corrected Snellen VA was converted to equivalent logarithm of the minimum angle of resolution (logMAR) VA. Follow-up data of logMAR VA, color fundus photography, and AF imaging were compared with those at baseline.

Color fundus photography was performed with a TRC-50IA Retinal Fundus Camera (Topcon, Tokyo, Japan). Patients were divided into 1 of 6 fundus appearance groups based on the presence and location of central (macular) RPE atrophy and yellowish-white flecks (Table 1).

Autofluorescence images before 2009 were obtained with an HRA 2 (Heidelberg Engineering, Heidelberg, Germany; excitation light, 488 nm, barrier filter, 500 nm; field of view, 30×30°); imaging after 2009 was undertaken using the Spectralis with viewing module version 5.1.2.0 (Heidelberg Engineering; excitation light, 488 nm; barrier filter, 500 nm; fields of view, 30×30° and 55×55°)^37^. Patients were classified into 1 of 3 AF patterns, as previously described (Table 1).^6,36^

Spectral domain OCT was undertaken with the Spectralis with viewing module version 5.1.2.0. The HEYEX software interface (version 1.6.2.0; Heidelberg Engineering) was used for retinal thickness measurements.^6,37^ Central foveal thickness was defined as the distance between the inner retinal surface and the inner border of the RPE.^6^

Electrophysiologic assessment included full-field electroretinogram (ERG), and pattern ERG, recorded with gold foil electrodes. Protocols incorporated the recommendations of the International Society for Clinical Electrophysiology of Vision.^38,39^ Full-field ERGs were used to assess generalized rod and cone system function and included (i) dark-adapted dim flash 0.01 cd·s·m^−2^ (DA 0.01), (ii) dark-adapted bright flash 11.0 cd·s·m^−2^ (DA 11.0), (iii) light-adapted 3.0 cd·s·m^−2^ 30 Hz flicker ERG (LA 3.0 30 Hz), and (iv) light-adapted 3.0 cd·s·m^−2^ at 2 Hz (LA 3.0). The pattern ERG P50 component was used to assess macular function. All the components of the ERG and the pattern ERG P50 component were examined to classify patients into 1 of the 3 previously described electrophysiologic groups (Table 1).^5,35^

### Mutation Screening

Blood samples were collected in EDTA tubes and DNA was extracted with a Nucleon Genomic DNA extraction kit (BACC2; Tepnel Life Sciences, West Lothian, UK).^8^ All 50 exons and exon–intron boundaries of the *ABCA4* gene were amplified using Illumina Truseq Custom Amplicon protocol (Illumina, San Diego, CA), followed by sequencing on Illumina MiSeq platform.^8,22^ The next-generation sequencing reads were analyzed and compared with the reference genome GRCh37/hg19, using the variant discovery software NextGENe (SoftGenetics LLC, State College, PA). All detected possibly disease-associated variants were confirmed by Sanger sequencing.^8,22^

All the missense variants identified were analyzed using 2 software prediction programs: SIFT (Sorting Intolerant from Tolerant; available from www.sift.jcvi.org/; accessed November 1, 2013), and PolyPhen2 (available from www.genetics.bwh.harvard.edu/pph/index.html; accessed November 1, 2013). Predicted effects on splicing of all the missense and intronic variants were assessed with the Human Splicing finder program version 2.4.1 (available from www.umd.be/HSF/; accessed November 1, 2013). The allele frequency of all variants was estimated by reference to the Exome Variant Server (NHLBI Exome Sequencing Project, Seattle, WA; available from www.snp.gs.washington.edu/EVS/; accessed November 1, 2013).

Patients harboring ≥2 mutations were classified into 3 genotype groups based on mutation type: Group A included patients with ≥2 definitely or likely deleterious (severe) variants; group B included patients with 1 deleterious variant and ≥1 missense or in-frame insertion/deletion variants; and group C included individuals with ≥2 missense or in-frame insertion/deletion variants^10^ (Table 1). One disease-associated intronic change of unknown effect was treated as a deleterious allele owing to the associated severe clinical phenotype previously reported.^5,22^ It should be noted, however, that assigning severity (e.g., a deleterious effect) to a mutation was not always straightforward, especially for missense alleles and some variants in splice sites.

### Comparison Between Childhood-Onset and Adult-Onset STGD

To investigate differences between the patients with childhood-onset STGD and those with adult-onset STGD, clinical and molecular genetic data of patients with adult-onset STGD ascertained at Moorfields Eye Hospital were reviewed. The comparison group consisted of all patients who had adult-onset STGD (older than 17 years), and who had ≥2 disease-causing *ABCA4* variants.

Statistical analysis was performed using commercially available software: Excel Tokei 2010 (Social Survey Research Information Co., Ltd., Tokyo, Japan). The eye used for analysis was selected according to the Random Integer Generator (available from www.random.org/). The Mann–Whitney *U* test was applied to investigate the differences between the 2 groups (childhood-onset STGD vs adult-onset STGD) in terms of logMAR VA, and central foveal thickness. The chi square statistic was applied to investigate the association between selected categorical variables of childhood-onset and adult-onset disease, including fundus appearance, flecks (macular, peripheral, and no flecks), presence of pigmentation, AF pattern, electrophysiologic group, and genotype group. *P* <0.05 was considered to indicate statistical significance.

## Results

Forty-two unrelated patients with childhood-onset STGD were ascertained; the clinical findings are summarized in Table 2 (available at www.aaojournal.org). There were 22 female and 20 male patients. Eight (19%) were from consanguineous families. The median age of onset was 8.5 years (range, 3-16), and the median age at baseline examination was 12.0 years (range, 7–16). The median logMAR VA at baseline in all 42 patients was 0.74 in the right eye and 0.74 in the left eye (range, 0.10–1.30 and 0.12–1.40, respectively). The mean duration of disease at baseline was 2.0 years (range, 0–9). Follow-up data were available for logMAR VA, fundus photography, and AF imaging, in 24, 14, and 11 patients, respectively. The detailed changes in these parameters during follow-up are presented in Table 3 (available at www.aaojournal.org).

The median logMAR VA at baseline in the 24 patients that were monitored was 0.75 in the right eye and 0.75 in the left eye (range, 0.10–1.30 and 0.12–1.30, respectively); the median logMAR VA at follow-up was 1.00 in the right and 1.00 in the left eye (range, 0.05–1.40 and 0.20–1.60, respectively) at a median age of 15.0 years (range, 12–16). Fifteen patients (15/42; 36%) had logMAR or ≤1.0 VA in the better eye at baseline. Thirteen of 24 patients (54%) with available follow-up data had logMAR VA of ≤1.0 in the better eye at follow-up (range, 11–16). Follow-up data were available in 14 of 27 patients with VA better than logMAR 1.0 in the better eye at baseline; 6 (43%) had logMAR of ≤1.0 VA in the better eye at follow-up (range, 13–15).

**Figure 1:**
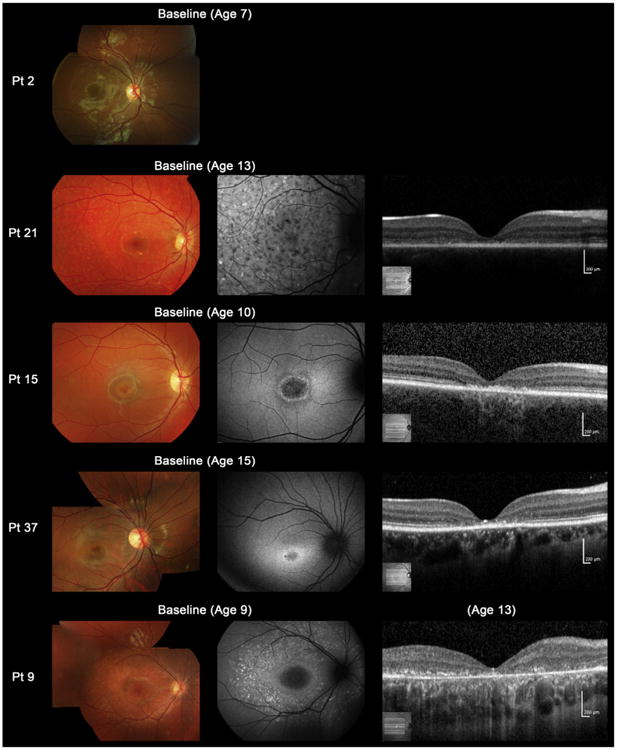
Color fundus photographs, autofluorescence, and spectral-domain optical coherence tomographic images of 5 representative cases with childhood-onset Stargardt Disease (patients 2, 21, 15, 37, and 9). Color fundus photographs of patient 2 shows normal findings at age 7 (fundus grade: 1). Patient 21 has numerous flecks at the posterior pole without central atrophy (fundus grade: 2) and autofluorescence (AF) imaging demonstrates widespread multiple foci of high and low AF signal at the posterior pole with a heterogeneous background (AF type 2). Spectral-domain optical coherence tomography (SD-OCT) identifies marked outer retinal loss at the central macula. Patient 15 has central atrophy without flecks (fundus grade: 3a) and AF imaging demonstrates a localized low AF signal at the fovea with a high signal edge surrounded by a homogeneous background (AF type: 1). SD-OCT detects marked outer retinal loss at the central macula. Patient 37 has central atrophy with macular flecks (fundus grade: 3b) and a localized low AF signal at the fovea surrounded by a homogeneous background with perifoveal foci of high signal (AF type: 1). SD-OCT shows outer retinal loss at the central macula. Patient 9 has central atrophy with peripheral flecks extending anterior to the vascular arcades (fundus grade: 3b) and a localized low AF signal at the macula surrounded by a heterogeneous background and widespread foci of high AF signal extending anterior to the vascular arcades (AF type: 2). SD-OCT reveals outer retinal disruption at the macula. Pt = patient.

Color fundus photographs, AF images, and SD-OCT images of 5 representative cases are shown in Figure 1. Baseline color fundus photographs were obtained in 39 patients (Table 2). Among the 39 patients, there was 1 (2.5%) with a grade 1 fundus appearance at baseline, 1 (2.5%) with grade 2, 11 (28%) with grade 3a, and 26 (67%) with grade 3b. There were no patients with a grade 3c or grade 4 fundus appearance. Central atrophy was present in 37 of the 39 patients (95%) at baseline; flecks were detected at the macula in 4 of the 39 patients (10%) and in the periphery in 23 (59%), with no visible flecks in 12 individuals (31%; Table 2). Retinal pigmentation was present in 2 of the 39 patients (5%; patients 24 and 34).

**Figure 2:**
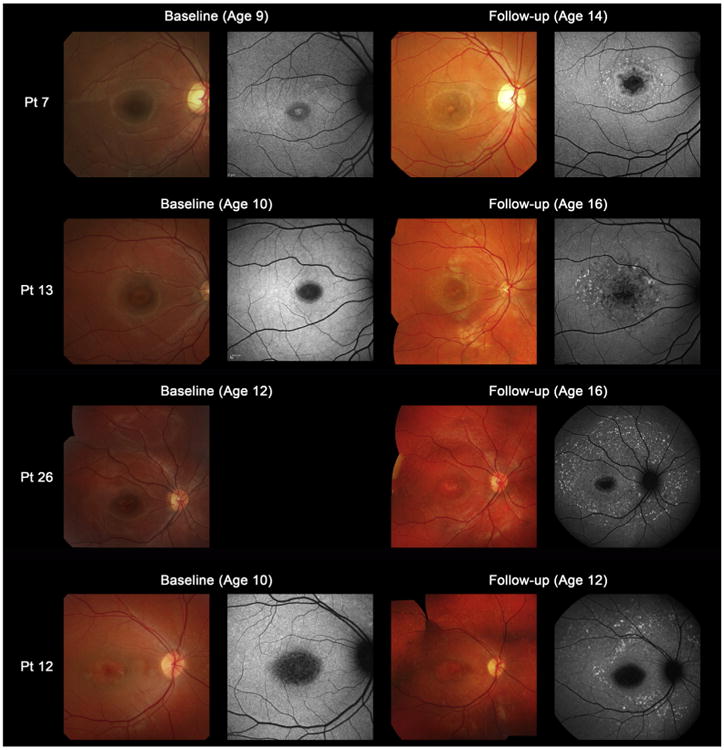
Color fundus photographs and autofluorescence (AF) images of 4 representative cases developing macular flecks during follow-up (patients 7, 13, 26, and 12). Color photograph of patient 7 at baseline shows subtle central atrophy without flecks (fundus grade 3a). At baseline, AF imaging demonstrates a localized low AF signal surrounded by an irregular high signal (AF type 1). Five years later, there is marked central atrophy with visible macular flecks (fundus grade 3b) and AF imaging demonstrates a localized low AF signal at the fovea with perifoveal foci of high signal (AF type 1). Patient 13 shows central atrophy with no visible flecks at baseline (fundus grade 3a), with AF imaging showing a localized low AF signal surrounded by subtle foci of high AF signal at the macula (AF type 1). Six years later, there are marked and increased macular flecks, also clearly seen on AF imaging (fundus grade 3b; AF type 1). Patient 26 has central atrophy with no visible flecks at baseline (fundus grade 3a), but marked flecks corresponding to foci of high signal on AF imaging are present 4 years later (fundus grade 3b; AF type 2). Patient 12 shows central atrophy with early subtle peripheral flecks at baseline (fundus grade 3b) and AF imaging demonstrates a localized low AF signal with subtle foci of high AF signal extending anterior to the vascular arcades (AF type 2). Two years later, there are marked and increased macular and peripheral flecks, which are also well-defined on AF imaging (fundus grade 3b; AF type 2). Pt = patient.

Serial color fundus photographs were available in 14 patients (Table 3), 3 of whom showed a fundus grade transition. Macular flecks, which were not present at baseline, developed in 2 subjects (patients 7 and 13) and macular and peripheral flecks became visible in 1 individual (patient 26). Color fundus photographs and AF images of 4 representative cases who developed flecks during the follow-up interval are shown in Figure 2.

**Figure 3:**
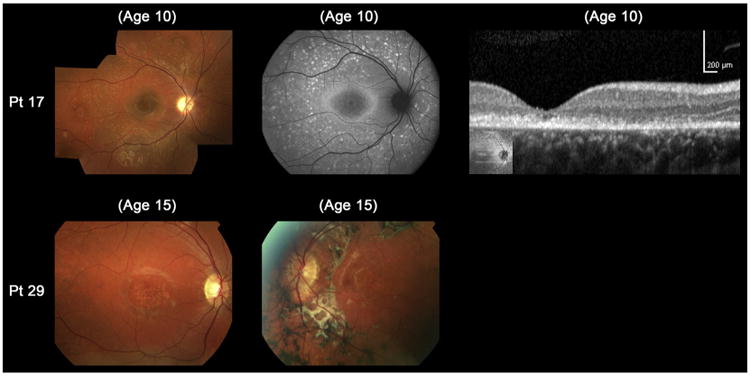
Color fundus photographs, autofluorescence (AF), and spectral-domain optical coherence tomographic images of 2 molecularly proven cases with “atypical” clinical features of childhood-onset Stargardt Disease (patients 17 and 29). Color photograph of patient 17 shows fine dots at the central macula surrounded by numerous peripheral flecks and AF imaging demonstrates well-defined dots associated with a high signal at the central macula surrounded by a ring of increased AF signal and numerous foci with high and low signal extending to the peripheral retina. Outer retinal loss at the macula is present on SD-OCT. Patient 29 has asymmetric fundus findings with central atrophy and peripheral flecks in the right eye and macular atrophy with flecks, subretinal fibrosis, and hyperpigmentation at the level of the retinal pigment epithelium in the left eye. Pt = patient.

Patients 17 and 18 had fine dots at the central macula surrounded by numerous peripheral flecks, classified into fundus grade 3b (patient 17; Fig 3). Clinical and molecular genetic data of these 2 patients have been previously described.^9^ Only 1 patient had asymmetric fundus findings, with a central atrophic-appearing lesion with peripheral flecks extending anterior to the vascular arcades in the right eye, and macular atrophy with flecks, subretinal fibrosis, and hyperpigmentation at the level of RPE in the left eye (patient 29; Fig 3).

We obtained AF images for 32 patients at baseline (Table 2). There were 10 of the 32 patients (31%) with type 1 AF pattern, 22 (69%) with type 2 AF, and no subjects with type 3 AF. Serial AF images were obtained in 11 patients during the follow-up interval (Table 3); no patient demonstrated an AF grouping transition.

We obtained SD-OCT images for 21 patients at baseline (Table 2). Outer retinal disruption at the fovea was present in all 21 patients. The median central foveal thickness of the right and left eyes was 60.0 and 55.0 μm, respectively (range, 33–138 and 35–140, respectively). Eighteen of the 21 patients (86%) had severe foveal thinning in both eyes (<100 μm).

Electrophysiologic assessment was performed in 25 patients at baseline (Table 2). Nine of the 25 patients (36%) were in ERG group 1 (isolated macular dysfunction),1 (4%) was in ERG group 2,and 15 (60%) were in ERG group 3 (generalized cone and rod dysfunction).

### Molecular Genetics

Detailed molecular genetic results including in silico analysis to assist in the prediction of pathogenicity of the variants are shown in Table 4 (available at www.aaojournal.org). Forty-six *ABCA4* variants were identified: 27 missense, 7 splice-site alterations, 7 nonsense, 3 frameshifts, 1 in-frame duplication, and 1 definitely disease-associated intronic variant for which the exact pathogenic mechanism is not known. Thirteen novel definitely or highly likely disease-causing variants were identified: p.Gln8fs, p.Cys519*, p.Asp586Gly, p.Arg587Lys, p.Glu905fs, p.Tyr1027*, p.Met1066-Arg, p.Arg1097*, p.Thr1721fs, p.Tyr1770Asp, p.Ala1739dup, p.Ser2072Asn, and c.6817-2A>C (Table 4). Four homozygous variants (p.Glu905fs, p.Glu1022Lys, p.Tyr1027*, and c.64719+1G>A) were identified in patients from consanguineous families and the other 42 variants were detected in heterozygous state. Four of 8 patients from consanguineous families had homozygous variants (patients 3, 5, 6, and 28), 2 had compound heterozygous variants (patients 2 and 17), and 2 had no variants identified (patients 1 and 4).

At least 1 disease-causing *ABCA4* variant was detected in 38 of the 42 patients (90%); of these, ≥2 variants were identified in 34 (81%) and 1 variant in 4 (9.5%; Tables 2 and 4). Only 4 of the 42 individuals (9.5%) had no variants identified. The 34 patients harboring ≥2 disease-causing variants were classified based on the number and mutation type (with suggested severity) into 3 genotype subgroups: 7 patients (21%) in genotype group A, 15 (44%) in group B, and 12 (35%) in group C (Table 2).

### Comparison Between Childhood-Onset and Adult-Onset STGD

**Figure 4:**
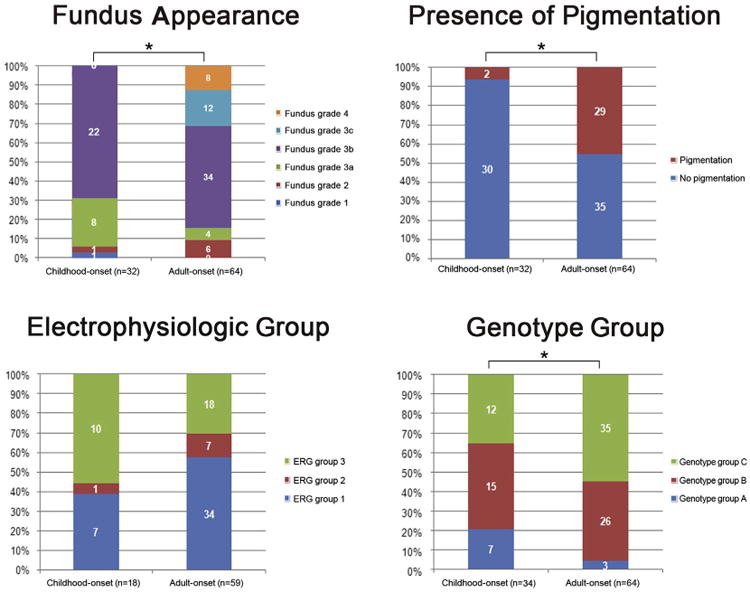
Comparison of the distribution of fundus appearances, presence of pigmentation, electrophysiologic group, and genotype group between a cohort with childhood-onset Stargardt disease and a group with adult-onset Stargardt disease. There are significant differences in terms of fundus appearance classification, presence of pigmentation, and genotype group classification (*P < 0.05). A higher proportion of patients with childhood-onset Stargardt disease are in electrophysiologic group 3 compared with adult-onset Stargardt disease, but this difference does not attain significance. ERG = electroretinography.

Sixty-four patients with adult-onset STGD harboring ≥2 disease-causing *ABCA4* variants were reviewed. The clinical and molecular genetic data were compared between 34 patients with childhood-onset STGD harboring ≥2 disease-causing *ABCA4* variants and the aforementioned 64 patients with adult-onset STGD (Table 5, available at www.aaojournal.org; Fig 4).

There were significant differences in terms of fundus appearance classification (chi-square = 23.2; *P* = 0.001), presence of pigmentation (chi-square = 14.9; *P* = 0.000), genotype group classification (chi-square = 7.3; *P* = 0.003), and central foveal thickness in the selected eye *(P* = 0.012; Table 5, available at www.aaojournal.org; Figs 4 and 5, available at www.aaojournal.org); with childhood-onset STGD being associated with less retinal pigmentation, a greater proportion of patients harboring deleterious alleles, and a thinner central fovea. No differences were identified in terms of location of flecks (chi-square = 4.0; *P* = 0.136), AF pattern (chi-square = 5.6; *P* = 0.061), electrophysiologic group (chi-square = 3.8; *P* = 0.148), or logMAR VA in the selected eye (*P* = 0.781). However, a greater proportion of patients with childhood-onset STGD were in ERG group 3 (10/18; 56%) compared with adult-onset STGD (18/59 [31%]; Table 5; Fig 4), but the difference, although showing a strong trend, did not attain significance (Fig 4).

## Discussion

This manuscript reports a series of childhood-onset patients with molecularly confirmed STGD, and compares the genetic, clinical, and electrophysiologic data with those in an adult-onset group.

The classical phenotype of STGD is characterized by the presence of yellowish-white fundus flecks and macular atrophy, but the fundus appearance can be variable.^1,3,12,13^ Fishman described 4 groups based on fundus appearance and electrophysiologic findings^3^; the author did not distinguish between childhood-onset and adult-onset disease. In addition, the classification did not fully encompass the range of phenotypes present in childhood-onset disease and thus was modified for the present study (Table 1). Most children in this study had the classical fundus appearance of STGD with macular atrophy and macular and/or peripheral flecks, but one third of children had no visible flecks at presentation. Subsequent development of flecks was observed during the follow-up interval in 3 of these 12 patients (Fig 2). Similar development of macular/peripheral flecks over time have also been described in a young adult patient with STGD.^18^

There were no children with paracentral atrophy without central atrophy (observed in the foveal sparing phenotype, a milder phenotype seen in a minority of patients with STGD). ^7,11,13,15^ This observation is in keeping with previous reports that patients with a foveal sparing phenotype typically present in later adult life.^7,15^ The subset with a foveal-sparing phenotype show relatively preserved foveal structure, which results in a relatively wide CFT range in the adult-onset STGD group (Fig 5).

Marked disruption of foveal outer retinal structure was present on SD-OCT in all children imaged, indicating that changes in foveal structure occur early in the disease process. Visual loss may precede ophthalmoscopic abnormalities in childhood-onset STGD and this may lead to nonorganic visual loss being considered. In such cases, SD-OCT imaging and/or electrophysiologic assessment will avoid misdiagnosis.^18^ The early foveal involvement in STGD without flecks, or other AF imaging evidence of increased levels of lipofuscin in the RPE, lend support to the hypothesis that A2E, which is elevated in STGD, may be directly toxic to cone photoreceptors.^40,41^

Of the 24 patients, 9 (36%) were in ERG group 1, 1 (4%) in ERG group 2, and 15 (60%) in ERG group 3. A greater proportion of patients were in group 3 compared with the cohort with adult-onset disease, indicating that childhood-onset STGD is more likely to be associated with generalized retinal dysfunction. This is further evidence for childhood-onset STGD having a more severe retinal phenotype. ^5,6,35^

Twenty-two patients (58%) had ≥1 deleterious variant and 7 subjects (18%) had 2 deleterious variants, which was significantly higher than observed in the adult-onset cohort (45% and 5%, respectively). The 5 patients (71%) with available ERGs in genotype group A (harboring 2 deleterious variants) all had generalized rod and cone system dysfunction (ERG group 3). These findings when taken together suggest that patients harboring deleterious *ABCA4* variants are more likely to have an earlier presentation (childhood) and a more severe functional phenotype.^5^

There are potential limitations of this study, including the definition of age of onset and choosing to classify childhood-onset as before the age of 17. The age of onset was defined as either the age at first symptom or the age when a retinal abnormality was first detected in “asymptomatic” patients. These 2 groups (symptomatic and asymptomatic) may have different clinical characteristics, including the symptomatic patients would be expected to have foveal involvement and thereby reduced VA. However, the vast majority of children were symptomatic in our cohort. It is also possible that dividing patients by age 17 may potentially introduce a selection bias.

This study specifically addresses, for the first time, the clinical features and molecular genetic findings of childhood-onset STGD in a substantial group of patients. Childhood-onset disease is associated with more severe VA loss from the early stages of disease. The classical flecks are not always present at diagnosis, but can appear later in the course of disease. Generalized cone and rod system dysfunction is more common than in adult-onset disease, in keeping with a more severe phenotype. Two or more disease-causing variants were detected in >80% of children and a higher proportion of definitely or possibly deleterious variants were demonstrated compared with adult-onset STGD, which is likely to underlie the earlier onset and more severe phenotype in childhood. The rapid deterioration of function in childhood-onset disease suggests that the investigation of novel therapies in this age group is more likely to lead to timely recognition of any treatment effect compared with adults with more slowly progressive disease.

## Supplementary Material

Figure 5 **Figure 5 Age of onset compared to logarithm of the minimum angle of resolution visual acuity and central foveal thickness for comparison between childhood-onset Stargardt disease and adult-onset Stargardt disease.** Scatter plots for the following parameters are shown; age of onset and logarithm of the minimum angle of resolution (logMAR) visual acuity, and age of onset and central foveal thickness (CFT) measured by spectral-domain optical coherence tomography (SD-OCT). The data of the selected eye of childhood-onset Stargardt disease (STGD) group are shown in blue and those from adult-onset STGD group shown in red. There was a significant difference detected by the Mann-Whitney U test between childhood-onset STGD and adult-onset STGD in terms of CFT; no significant difference was revealed in logMAR visual acuity.

Table 2

Table 3

Table 4

Table 5

## Figures and Tables

**Table 1:** Classification of Phenotype and Genotype in Stargardt Disease, Based on Fundus Appearance, Autofluorescence Pattern, Electrophysiologic Assessment, and *ABCA4* Variants

Fundus Appearance		AF Pattern		ERG Group		Genotype Group Classification	
Grade 1	Normal fundus	Type 1	Localized low AF signal at the fovea surrounded by a homogeneous background with/without perifoveal foci of high or low signal	Group 1	PERG abnormality with normal full-field ERGs	Genotype A	Two or more (likely) deleterious variants
Grade 2	Macular and/or peripheral flecks without central atrophy						
Grade 3a	Central atrophy without flecks	Type 2	Localized low AF signal at the macula surrounded by a heterogeneous background and widespread foci of high or low AF signal extending anterior to the vascular arcades	Group 2	PERG abnormality with additional generalized cone ERG abnormality	Genotype B	One deleterious variant and ≥1 missense or inframe insertion/deletion variant(s)
Grade 3b	Central atrophy with macular and/or peripheral flecks						
Grade 3c	Paracentral atrophy with macular and/or peripheral flecks, without central atrophy						
Grade 4	Multiple extensive atrophic changes of the RPE, extending beyond the vascular arcades	Type 3	Multiple areas of low AF signal at posterior pole with a heterogeneous background and/or foci of high or low signal	Group 3	PERG abnormality with additional generalized cone and rod ERG abnormality	Genotype C	Two or more missense or in-frame insertion/deletion variants

AF = autofluorescence; ERG = electroretinography; PERG = pattern electroretinography; RPE = retinal pigment epithelium.

Aligned grades/types/groups of 4 classifications do not correlate with each other.

**Table 2:** Summary of Clinical Findings at Baseline and Molecular Status of 42 patients with Childhood-onset Stargardt Disease

							OCT				
Pt	Onset (yrs)	Age at Baseline (yrs)	LogMAR VA		Fds type	AF type	CFT (μm)		ERG group	Genotype group	Mutation Status
			R	L			R	L			
1*	4	6	1.00	0.78	3b	NA	NA	NA	3	NA	No variants
2*	6	7	0.48	0.48	1	NA	NA	NA	1	B	p.[Arg653Cys];[Arg2030*]
3*	7	7	1.30	1.20	3b	2	60	45	3	A	c.[6479+1G>A];[6479+1G>A]
4*	3	7	0.10	0.20	3b	NA	NA	NA	3	NA	No variants
5*	5	8	1.00	1.00	3b	2	NA	NA	3	A	**p.[Glu905fs];[Glu905fs]**
6	7	8	1.30	1.40	3b	2	49	44	NA	A	p.[Arg1097*];c.[5196+1G>A]
7	7	8	0.48	0.40	3a	1	46	38	1	B	p.[Arg212Cys];c.[5461-10T>C]
8*	6	8	0.48	0.48	3a	2	NA	NA	3	A	p.[Tyr1027*];[Tyr1027*]
9	7	9	0.60	0.20	3b	2	61	67	NA	B	p.[Cys1490Tyr];c.[5461-10T>C]
10	8	9	0.70	0.70	3b	2	69	65	3	B	p.[Glu1087Lys];c.[5461-10T>C]
11	9	9	0.48	0.48	3b	2	NA	NA	3	C	p.[Arg1108Cys];[Cys1490Tyr]
12	8	9	1.00	1.00	3b	2	NA	NA	1	NA	p.Cys519*
13	7	9	0.48	0.48	3a	1	35	45	NA	C	p.[Arg1108Cys];[Thr1526Met]
14	8	9	0.60	0.48	3b	2	NA	NA	1	C	p.[His1406Tyr];[Trp1408Arg;Arg1640Trp]
15	5	10	0.80	0.80	3a	1	54	54	3	NA	c.5461-10T>C
16	8	10	1.00	1.00	3b	2	NA	NA	NA	B	p.[Tyr954Ser];c.[5461-10T>C]
17*†	5	10	0.30	0.30	3b	2	72	81	NA	B	c.[768G>T];p.[Cys1455Arg]
18†	9	11	0.50	0.40	3b	2	94	107	3	A	p.[Gln636*];c.[5461-10T>C]
19	9	11	0.30	0.30	3a	NA	NA	NA	3	NA	No variants
20	10	11	0.78	0.78	3b	2	61	66	3	A	**p.[Gln8fs];**c.[5461-10T>C]
21	10	11	0.54	0.12	2	2	61	70	1	B	p.[Trp439*];[Pro1380Leu]
22	8	11	1.00	1.00	3b	2	NA	NA	NA	B	c.[5461-10T>C];p.[Leu2027Phe]
23	12	12	0.18	0.18	3b	NA	NA	NA	1	NA	No variants
24	11	12	1.00	0.90	3b	2	37	41	NA	B	p.[Cys1455Arg];c.[5714+5G>A]
25	8	12	1.30	1.30	3b	2	78	84	NA	B	p.[Gly863Ala(;)Glu1122Lys(;)Arg2030*]
26	3	12	1.00	1.00	3a	2	NA	NA	NA	C	p.[Gly550Arg];[Cys2150Tyr]
27	9	13	0.50	0.60	3a	1	138	140	1	B	p.[Gly863Ala];**[Thr1721fs]**
28*	6	13	1.30	1.10	3b	2	33	35	3	C	p.[Glu1022Lys];[Glu1022Lys]
29	8	13	1.00	1.18	3b	NA	NA	NA	NA	B	**p.[Arg587Lys]**;[Trp855*]
30	11	14	0.90	0.80	3b	2	73	67	NA	A	p.[Gln636*];**c.[6817-2A**>**C**]
31	12	14	0.48	0.48	3a	1	NA	NA	1	C	p.[Thr1019Met];[Gly1961Glu]
32	10	14	1.00	1.00	NA	NA	NA	NA	3	B	c.[5018+2T>C];**p.[Ser2072Asn**]
33	11	14	0.18	0.20	3a	1	126	134	1	B	p.[Gly1961Glu];c.[6729+4_6729+18del]
34	12	15	1.00	1.00	3b	2	48	49	3	C	p.[Pro1380Leu]; **[Tyr1770Asp]**
35	12	15	0.50	0.50	3a	1	46	50	3	NA	p.Gly1961Glu
36	12	15	1.00	1.00	3b	2	60	55	2	C	p.[Arg653Cys];[Pro1380Leu]
37	14	15	0.18	0.18	3b	1	44	51	NA	C	p.[Arg511Cys(;)**Ala1739dup**(;)Gly1961Glu]
38	13	15	1.00	1.00	3b	NA	NA	NA	NA	C	**p.[Met1066Arg]**;[Cys1490Tyr]
39	12	15	0.80	0.80	3b	1	NA	NA	NA	C	**p.[Asp586Gly]**;[Gly1961Glu]
40	16	16	0.48	0.48	NA	NA	NA	NA	NA	NA	p.Leu2027Phe
41	13	16	1.00	1.00	NA	NA	NA	NA	NA	B	c.[5461-10T>C];p.[Leu2027Phe]
42	14	16	0.18	0.18	3a	1	NA	NA	NA	C	p.[Arg1129Cys];[Cys1490Tyr]

AF type = autofluorescence type; CFT = central foveal thickness; ERG = electroretinogram; Fds type = fundus type; L = left; LogMAR VA = logarithm of the minimum angle of resolution visual acuity; NA = not available; OCT = optical coherence tomography; Pt = patient; R = right.

The age of onset was defined as either the age at which visual loss was first noted by the patient or in the asymptomatic patients when abnormal retinal appearance was first detected.

The CFT was defined as the distance between the inner retinal surface and inner border of the retinal pigment epithelium at the central fovea.

* Eight patients were from consanguineous families.

† Two patients have been partially described in a previous case report (patients 17 and 18).^9^

Variants shown in bold are putative novel.

**Table 3:** Detailed Changes of Visual Acuity, Fundus Appearance, Autofluorescence Pattern during the Follow-up Interval of 42 patients with Childhood-onset Stargardt Disease

	LogMAR VA						Fds type					AF type				
Pt		BL			FU		BL		FU		Type transision	BL		FU		Type transision
	Age (yrs)	R	L	Age (yrs)	R	L	Age (yrs)		Age (yrs)			Age (yrs)		Age (yrs)		
1	6	1.00	0.78		NA		6	3b	13	3b		NA		NA		
2	7	0.48	0.48		NA		7	1	NA			NA		NA		
3	7	1.30	1.20		NA		9	3b	10	3b		9	2	10	2	
4	7	0.10	0.20	12	0.05	0.20	10	3b	12	3b		NA		NA		
5	8	1.00	1.00		NA		9	3b	NA			9	2	NA		
6	8	1.30	1.40		NA		11	3b	NA			11	2	NA		
7	8	0.48	0.40	14	0.80	0.70	9	3a	14	3b	✓	9	1	14	1	
8	8	0.48	0.48	15	1.40	1.60	16	3a	NA			16	2	NA		
9	9	0.60	0.20	13	1.10	1.00	9	3b	12	3b		9	2	13	2	
10	9	0.70	0.70	13	1.10	1.20	9	3b	NA			9	2	13	2	
11	9	0.48	0.48	14	1.30	1.20	9	3b	13	3b		9	2	13	2	
12	9	1.00	1.00	14	1.10	0.90	10	3b	13	3b		10	2	12	2	
13	9	0.48	0.48	16	0.80	0.80	10	3a	14	3b	✓	10	1	16	1	
14	9	0.60	0.48	16	1.00	1.00	10	3b	NA			10	2	NA		
15	10	0.80	0.80		NA		10	3a	NA			10	1.00	NA		
16	10	1.00	1.00	11	1.00	1.10	11	3b	NA			11	2	NA		
17	10	0.30	0.30	14	0.30	0.30	10	3b	NA			10	2	NA		
18	11	0.50	0.40		NA		11	3b	NA			11	2	NA		
19	11	0.30	0.30		NA		11	3a	NA			NA		NA		
20	11	0.78	0.78		NA		11	3b	12	3b		11	2	12	2	
21	11	0.54	0.12	13	0.80	0.30	13	2	NA			13	2	NA		
22	11	1.00	1.00	16	1.00	NA	13	3b	15	3b		15	2	NA		
23	12	0.18	0.18		NA		16	3b	NA			NA		NA		
24	12	1.00	0.90	15	1.00	1.00	12	3b	15	3b		12	2	15	2	
25	12	1.30	1.30	16	1.30	1.18	12	3b	15	3b		13	2	16	2	
26	12	1.00	1.00	16	1.00	1.00	12	3a	16	3b	✓	16	2	NA		
27	13	0.50	0.60		NA		13	3a	NA			13	1	NA		
28	13	1.30	1.10	15	1.10	0.88	14	3b	NA			14	2	15	2	
29	13	1.00	1.18	16	0.78	0.78	15	3b	NA			NA		NA		
30	14	0.90	0.80		NA		15	3b	NA			15	2	NA		
31	14	0.48	0.48		NA		14	3a	NA			14	1	NA		
32	14	1.00	1.00	15	1.00	1.00	NA		NA			NA		NA		
33	14	0.18	0.20	16	0.36	0.36	16	3a	NA			16	1	NA		
34	15	1.00	1.00		NA		15	3b	NA			15	2	NA		
35	15	0.50	0.50		NA		15	3a	NA			15	1	NA		
36	15	1.00	1.00	16	1.00	1.00	16	3b	NA			16	2	NA		
37	15	0.18	0.18	16	0.75	0.56	15	3b	16	3b		16	1	NA		
38	15	1.00	1.00	16	1.08	1.00	16	3b	NA			NA		NA		
39	15	0.80	0.80	16	0.80	0.80	15	3b	NA			15	1	NA		
40	16	0.48	0.48		NA		NA		NA			NA		NA		
41	16	1.00	1.00		NA		NA		NA			NA		NA		
42	16	0.18	0.18		NA		16	3a	NA			16	1	NA		

AF type = autofluorescence type; BL = baseline; Fds type = fundus type; FU = follow-up; L = left; NA; LogMAR VA = logarithm of the minimum angle of resolution visual acuity; NA = not available; Pt = patient; R = right;

**Table 4:** Suggested Pathogenicity of the 46 *ABCA4* Variants Identified in Childhood-onset Stargardt Disease

Exon/ IVS	Nucleotide substitution	Protein change/effect	Number of alleles identified	Pt	Reference	SIFT	Polyphen2		HSF			Allelic frequency observed by EVS	db SNP
						Prediction	Prediction	Hum var score (0-1)	Wild type CV	Mutant CV	effect		
1	c.21dupA	p.Gln8fs	1	20	This study							ND	
6	c.634C>T	p.Arg212Cys	1	7	Simonelli F et al.^30^	Not tolerated	PRD	0.951				0.0116	rs61750200
6	c.768G>T	Splice	1	17	Klevering et al.^28^				91.6	80.7	weakens the splice donor site by ∼12%	ND	
10	c.1317G>A	p.Trp439*	1	21	Fujinami et al.^5^							ND	
11	c.1531C>T	p.Arg511Cys	1	37	Zernant et al.^22^	Not tolerated	PRD	0.976				ND	
12	c.1557C>A	p.Cys519*	1	12	This study							ND	
12	c.1648G>A	p.Gly550Arg	1	26	Shroyer et al.^27^	Not tolerated	POD	0.882	0	81.58	creates a new splice acceptor site	ND	
12	c.1757A>G	p.Asp586Gly	1	39	This study	Not tolerated	POD	0.599				ND	
12	c.1760G>A	p.Arg587Lys	1	29	This study	Not tolerated	POD	0.749	84.6	74	weakens the splice donor site by ∼13%	ND	
13	c.1906C>T	p.Gln636*	3	2, 18, 30	Zernant et al.^22^							0.0116	rs145961131
14	c.1957C>T	p.Arg653Cys	1	36	Rivera et al.^25^	Not tolerated	PRD	0.999				ND	
16	c.2564G>A	p.Trp855*	1	29	Rivera et al.^25^								rs61752406
17	c.2588G>C	p.Gly863Ala/ p.Gly863del	2	25, 27	Lewis et al.^24^/Maugeri et al.^34^	Not tolerated	POD	0.864				0.6744	rs76157638
18	c.2712delG	p.Glu905fs	2	5	This study							ND	
19	c.2861A>C	p.Tyr954Ser	1	16	Aguirre-Lamban et al.^32^	Not tolerated	PRD	0.959				ND	
21	c.3056C>T	p.Thr1019Met	1	31	Rozet et al.^23^	Not tolerated	PRD	1.000				ND	rs201855602
21	c.3064G>A	p.Glu1022Lys	2	28	Webster et al.^26^	Not tolerated	PRD	1.000				ND	rs61749459
21	c.3081T>G	p.Tyr1027*	2	8	This study							ND	
22	c.3197T>G	p.Met1066Arg	1	38	This study	Not tolerated	POD	0.495				ND	
22	c.3259G>A	p.Glu1087Lys	1	10	Lewis et al. 1999	Not tolerated	PRD	0.997				ND	rs61751398
22	c.3289A>T	p.Arg1097*	1	6	This study							ND	
22	c.3322C>T	p.Arg1108Cys	2	11, 13	Rozet et al.^23^	Not tolerated	PRD	0.986				0.0116	rs61750120
23	c.3364G>A	p.Glu1122Lys	1	25	Lewis et al.^24^	Not tolerated	PRD	1.000				ND	rs61751399
23	c.3385C>T	p.Arg1129Cys	1	42	Zernant et al.^22^	Not tolerated	PRD	0.998				ND	
28	c.4139C>T	p.Pro1380Leu	3	21, 34, 36	Lewis et al.^24^	Not tolerated	PRD	0.99				0.0233	rs61750130
28	c.4216C>T	p.His1406Tyr	1	14	Lewis et al.^24^	Not tolerated	POD	0.824				ND	rs61750133
28	c.4222T>C	p.Trp1408Arg	1	14	Lewis et al.^24^	Not tolerated	PRD	0.973				ND	rs61750135
30	c.4363T>C	p.Cys1455Arg	2	17, 24	Fujinami et al.^5^	Not tolerated	PRD	0.999				ND	
30	c.4469G>A	p.Cys1490Tyr	4	9, 11, 38, 42	Lewis et al.^24^	Not tolerated	PRD	0.994				ND	rs61751402
31	c.4577C>T	p.Thr1526Met	1	13	Lewis et al.^24^	Not tolerated	PRD	0.999				ND	rs61750152
36	c.4918C>T	p.Arg1640Trp	1	14	Briggs et al.^22^	Not tolerated	PRD	0.999				ND	
36	c.5160_5161delCA	p.Thr1721fs	1	27	This study							ND	rs61750566
37	c.5308T>G	p.Tyr1770Asp	1	34	This study	Not tolerated	PRD	1.000				ND	
37	c.5213_5214insTGC	p.Ala1739dup	1	37	This study							ND	
42	c.5882G>A	p.Gly1961Glu	5	31, 33, 35, 37, 39	Lewis et al.^24^	Not tolerated	PRD	1.000				0.4186	rs1800553
44	c.6079C>T	p.Leu2027Phe	3	22, 40, 41	Lewis et al.^24^	Not tolerated	PRD	1.000				0.0349	rs61751408
44	c.6088C>T	p.Arg2030*	2	2, 25	Lewis et al.^24^							ND	rs61751383
45	c.6215G>A	p.Ser2072Asn	1	32	This study	Not tolerated	PRD	1.000				ND	
47	c.6449G>A	p.Cys2150Tyr	1	26	Fishman et al.^16^	Not tolerated	PRD	1.000				0.0116	rs61751384
IVS35	c.5018+2T>C	splice	1	32	Fujinami et al.^8^				81.15	0	eliminates the splice donor site	ND	
IVS36	c.5196+1G>A	splice	1	6	Shroyer et al.^27^				83.28	0	eliminates the splice donor site	ND	
IVS38	c.5461-10T>C	Uncertain	9	7, 9, 10, 15, 16, 18, 20, 22, 41	Briggs et al.^20^							0.0349	rs1800728
IVS40	c.5714+5G>A	splice	1	24	Cremers et al.^19^				85.49	73.33	weakens the splice donor site by ∼14%	0.1512	
IVS47	c.6479+1G>A	splice	2	3	Zernant et al.^22^				87.25	0	eliminates the splice donor site	ND	
IVS48	c.6729+4_6729+18d elAGTTGGCCCTG GGGC	splice	1	33	Littink et al.^31^							ND	
IVS49	c.6817-2A>C	splice	1	30	This study				93.6	0	eliminates the splice acceptor site	ND	

CV = consensus value; EVS = Exon variant server; Het = heterozygous; Hom = homozygous; HSF = human splicing finder; Hum Var Score = human var score; IVS = intervening sequence; NA = not applicable; ND= not detected; POD = possibly damaging; PRD = probably damaging; Pt = patient; SIFT = Sorting Intolerant From Tolerant ; WT = wild type.

SIFT (version 4.0.4) results are reported to be tolerant if tolerance index ≥ 0.05 or intolerant if tolerance index < 0.05. [http://sift.bii.a-star.edu.sg/www/SIFT_BLink_submit.html/. Accessed February 1, 2013.] Polyphen 2 (vision 2.1) appraises mutations qualitatively as Benign, Possibly Damaging or Probably Damaging based on the model's false positive rate. [http://genetics.bwh.harvard.edu/pph2/. Accessed November 1, 2013.] HumanVar-trained model of Polyphen 2 was selected, since diagnostics of mendelian diseases requires distinguishing mutations with drastic effects from all the remaining human variation, including abundant mildly deleterious alleles. The cDNA is numbered according to Ensemble transcript ID ENST00000370225, in which +1 is the A of the translation start codon. Human Splicing Finder (HSF, version 2.4.1) reports the results from the HSF matrix: the higher the consensus value, the stronger the predicted splice site. The values for the wildtype and mutant sequences are shown; the larger the difference between these values, the greater the chance that the variant can affect splicing [http://www.umd.be/HSF/. Accessed November 1, 2013.]. EVS denotes the allele frequencies of variants on the Exome Variant Server, NHLBI Exome Sequencing Project, Seattle, WA, USA. [http://snp.gs.washington.edu/EVS/. Accessed Febrary 1, 2013.]

**Table 5:** Comparison of Clinical Characteristics, Genotype, and Allele Frequency of the Prevalent Variants between Childhood-onset and Adult-onset Stargardt Disease with two or more disease-causing *ABCA4* variants

	Childhood-onset Stargardt disease (n=34)						Adult-onset Stargardt disease (n=64)					
Median age of onset (yrs)	8.5 (3-14)						27.0 (17-65)					
Median age at examination (yrs)	11.5 (7-16)						44.0 (21-71)					
Median LogMAR VA of the right eye	0.79 (0.18-1.30)						1.00 (-0.08-2.00)					

Fundus Appearance	Total (n=32)						Total (n=64)					
	Grade 1	Grade 2	Grade 3a	Grade 3b	Grade 3c	Grade 4	Grade 1	Grade 2	Grade 3a	Grade 3b	Grade 3c	Grade 4
	1	1	8	22	0	0	0	6	4	34	12	8
		Flecks			No flecks			Flecks			No flecks	
	Macular		Peripheral				Macular		Peripheral			
	2		21		9		14		39		11	
		Pigmentation			No pigmentation			Pigmentation			No pigmentation	
		2			30			29			35	

Autofluorescence Pattern	Total (n=29)						Total (n=62)					
	Type 1		Type 2		Type 3		Type 1		Type 2		Type 3	
	8		21		0		20		33		9	

OCT, CFT (μm) of the right eye	Total (n=19)						Total (n=33)					
	61.0 (33-138)						81.0 (20-297)					

ERG group	Total (n=18)						Total (n=59)					
	Group 1		Group 2		Group 3		Group 1		Group 2		Group 3	
	7		1		10		34		7		18	

Genotype group classification	Total (n=34)						Total (n=64)					
	Group A		Group B		Group C		Group A		Group B		Group C	
	7		15		12		3		26		35	

Frequencies of the most prevalent variants	c.5461-10T>C		p.Gly1961Glu		p.Cys1490Tyr		p.Gly1961Glu		p.Gly863Ala		p.Leu2027Phe	
	8 (11.8%)		4 (5.9%)		4 (5.9%)		16 (12.5%)		13 (10.1%)		8 (6.3%)	

AF type = autofluorescence type; CFT = central foveal thickness; ERG = electroretinogram; LogMAR VA = logarithm of the minimum angle of resolution visual acuity; OCT = optical coherence tomography.

In order to investigate the differences between the patients with childhood-onset Stargardt Disease (STGD) and those with adult-onset STGD, clinical and molecular genetic data of patients with adult-onset STGD ascertained at Moorfields Eye Hospital were reviewed. The comparison group consisted of all patients who had adult-onset STGD (older than 17 years old), and two or more disease-causing *ABCA4* variants. For the purpose of this comparison, 34 patients with childhood-onset STGD and two or more disease-causing *ABCA4* variants were selected.
