# Supplementary material for: Clinical and Molecular Characteristics of Childhood-Onset Stargardt Disease
Source: Ophthalmology. Author manuscript; Available in PMC 2015 Jun 8. (PMC4459618; doi:10.1016/j.ophtha.2014.08.012)
Supplement: Figure 5 — Age of onset compared to logarithm of the minimum angle of resolution visual acuity and central foveal thickness for comparison between childhood-onset Stargardt disease and adult-onset Stargardt disease. Scatter plots for the following parameters are shown; age of onset and logarithm of the minimum angle of resolution (logMAR) visual acuity, and age of onset and central foveal thickness (CFT) measured by spectral-domain optical coherence tomography (SD-OCT). The data of the selected eye of childhood-onset Stargardt disease (STGD) group are shown in blue and those from adult-onset STGD group shown in red. There was a significant difference detected by the Mann-Whitney U test between childhood-onset STGD and adult-onset STGD in terms of CFT; no significant difference was revealed in logMAR visual acuity. [file NIHMS693250-supplement-Figure_5.pdf]

Onset vs logMAR visual acuity

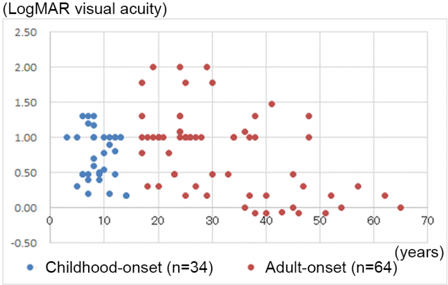

Onset vs central foveal thickness

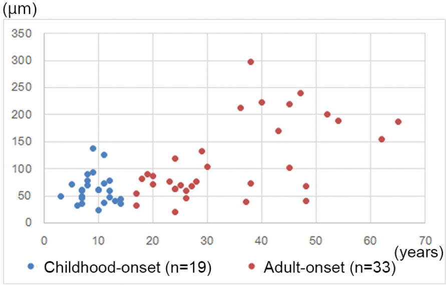

Supplemental Figure Legends

Figure 5 Age of onset compared to logarithm of the minimum angle of resolution visual acuity and central foveal thickness for comparison between childhood-onset Stargardt disease and adult-onset Stargardt disease.

Scatter plots for the following parameters are shown; age of onset and logarithm of the minimum angle of resolution (logMAR) visual acuity, and age of onset and central foveal thickness (CFT) measured by spectral-domain optical coherence tomography (SD-OCT). The data of the selected eye of childhood-onset Stargardt disease (STGD) group are shown in blue and those from adult-onset STGD group shown in red. There was a significant difference detected by the Mann-Whitney U test between childhood-onset STGD and adult-onset STGD in terms of CFT; no significant difference was revealed in logMAR visual acuity.
