# Supplementary material for: Clinical and Molecular Characteristics of Childhood-Onset Stargardt Disease
Source: Ophthalmology. Author manuscript; Available in PMC 2015 Jun 8. (PMC4459618; doi:10.1016/j.ophtha.2014.08.012)
Supplement: Table 2 [file NIHMS693250-supplement-Table_2.pdf]

Table 2. Summary of Clinical Findings at Baseline and Molecular Status of 42 patients with Childhood-onset Stargardt Disease

Pt	Onset (yrs)	Age at Baseline (yrs)	LogMAR VA		Fds type	AF type	OCT CFT (μm)		ERG group	Genotype group	Mutation Status
			R	L			R	L			
1*	4	6	1.00	0.78	3b	NA	NA	NA	3	NA	No variants
2*	6	7	0.48	0.48	1	NA	NA	NA	1	B	p.[Arg653Cys];[Arg2030*]
3*	7	7	1.30	1.20	3b	2	60	45	3	A	c.[6479+1G>A];[6479+1G>A]
4*	3	7	0.10	0.20	3b	NA	NA	NA	3	NA	No variants
5*	5	8	1.00	1.00	3b	2	NA	NA	3	A	**p.[Glu905fs];[Glu905fs]**
6	7	8	1.30	1.40	3b	2	49	44	NA	A	**p.[Arg1097*];**c.[5196+1G>A]
7	7	8	0.48	0.40	3a	1	46	38	1	B	p.[Arg212Cys];c.[5461-10T>C]
8*	6	8	0.48	0.48	3a	2	NA	NA	3	A	**p.[Tyr1027*];[Tyr1027*]**
9	7	9	0.60	0.20	3b	2	61	67	NA	B	p.[Cys1490Tyr];c.[5461-10T>C]
10	8	9	0.70	0.70	3b	2	69	65	3	B	p.[Glu1087Lys];c.[5461-10T>C]
11	9	9	0.48	0.48	3b	2	NA	NA	3	C	p.[Arg1108Cys];[Cys1490Tyr]
12	8	9	1.00	1.00	3b	2	NA	NA	1	NA	**p.Cys519***
13	7	9	0.48	0.48	3a	1	35	45	NA	C	p.[Arg1108Cys];[Thr1526Met]
14	8	9	0.60	0.48	3b	2	NA	NA	1	C	p.[His1406Tyr];[Trp1408Arg;Arg1640Trp]
15	5	10	0.80	0.80	3a	1	54	54	3	NA	c.5461-10T>C
16	8	10	1.00	1.00	3b	2	NA	NA	NA	B	p.[Tyr954Ser];c.[5461-10T>C]
17*[†]	5	10	0.30	0.30	3b	2	72	81	NA	B	c.[768G>T];p.[Cys1455Arg]
18[†]	9	11	0.50	0.40	3b	2	94	107	3	A	p.[Gln636*];c.[5461-10T>C]

19	9	11	0.30	0.30	3a	NA	NA	NA	3	NA	No variants
20	10	11	0.78	0.78	3b	2	61	66	3	A	**p.[Gln8fs];**c.[5461-10T>C]
21	10	11	0.54	0.12	2	2	61	70	1	B	p.[Trp439*];[Pro1380Leu]
22	8	11	1.00	1.00	3b	2	NA	NA	NA	B	c.[5461-10T>C];p.[Leu2027Phe]
23	12	12	0.18	0.18	3b	NA	NA	NA	1	NA	No variants
24	11	12	1.00	0.90	3b	2	37	41	NA	B	p.[Cys1455Arg];c.[5714+5G>A]
25	8	12	1.30	1.30	3b	2	78	84	NA	B	p.[Gly863Ala(;)Glu1122Lys(;)Arg2030*]
26	3	12	1.00	1.00	3a	2	NA	NA	NA	C	p.[Gly550Arg];[Cys2150Tyr]
27	9	13	0.50	0.60	3a	1	138	140	1	B	p.[Gly863Ala];**[Thr1721fs]**
28*	6	13	1.30	1.10	3b	2	33	35	3	C	p.[Glu1022Lys];[Glu1022Lys]
29	8	13	1.00	1.18	3b	NA	NA	NA	NA	B	**p.[Arg587Lys]**;[Trp855*]
30	11	14	0.90	0.80	3b	2	73	67	NA	A	p.[Gln636*];**c.[6817-2A>C**]
31	12	14	0.48	0.48	3a	1	NA	NA	1	C	p.[Thr1019Met];[Gly1961Glu]
32	10	14	1.00	1.00	NA	NA	NA	NA	3	B	c.[5018+2T>C];**p.[Ser2072Asn**]
33	11	14	0.18	0.20	3a	1	126	134	1	B	p.[Gly1961Glu];c.[6729+4_6729+18del]
34	12	15	1.00	1.00	3b	2	48	49	3	C	p.[Pro1380Leu];**[Tyr1770Asp]**
35	12	15	0.50	0.50	3a	1	46	50	3	NA	p.Gly1961Glu
36	12	15	1.00	1.00	3b	2	60	55	2	C	p.[Arg653Cys];[Pro1380Leu]
37	14	15	0.18	0.18	3b	1	44	51	NA	C	p.[Arg511Cys(;)**Ala1739dup**(;)Gly1961Glu]
38	13	15	1.00	1.00	3b	NA	NA	NA	NA	C	**p.[Met1066Arg]**;[Cys1490Tyr]
39	12	15	0.80	0.80	3b	1	NA	NA	NA	C	**p.[Asp586Gly]**;[Gly1961Glu]
40	16	16	0.48	0.48	NA	NA	NA	NA	NA	NA	p.Leu2027Phe
41	13	16	1.00	1.00	NA	NA	NA	NA	NA	B	c.[5461-10T>C];p.[Leu2027Phe]

42	14	16	0.18	0.18	3a	1	NA	NA	NA	C	p.[Arg1129Cys];[Cys1490Tyr]

AF type = autofluorescence type; CFT = central foveal thickness; ERG = electroretinogram; Fds type = fundus type; L = left; LogMAR VA = logarithm of the minimum angle of resolution visual acuity; NA = not available; OCT = optical coherence tomography; Pt = patient; R = right.

The age of onset was defined as either the age at which visual loss was first noted by the patient or in the asymptomatic patients when abnormal retinal appearance was first detected.

The CFT was defined as the distance between the inner retinal surface and inner border of the retinal pigment epithelium at the central fovea.

* Eight patients were from consanguineous families.

†Two patients have been partially described in a previous case report (patients 17 and 18).[9]

Variants shown in bold are putative novel.
