# Supplementary material for: Clinical and Molecular Characteristics of Childhood-Onset Stargardt Disease
Source: Ophthalmology. Author manuscript; Available in PMC 2015 Jun 8. (PMC4459618; doi:10.1016/j.ophtha.2014.08.012)
Supplement: Table 3 [file NIHMS693250-supplement-Table_3.pdf]

Table 3. Detailed Changes of Visual Acuity, Fundus Appearance, Autofluorescence Pattern during the Follow-up Interval of 42 patients with Childhood-onset Stargardt Disease

Pt	LogMAR VA						Fds type					AF type				
	BL			FU			BL		FU		Type transision	BL		FU		Type transision
	Age (yrs)	R	L	Age (yrs)	R	L	Age (yrs)		Age (yrs)			Age (yrs)		Age (yrs)		
1	6	1.00	0.78		NA		6	3b	13	3b		NA		NA		
2	7	0.48	0.48		NA		7	1	NA			NA		NA		
3	7	1.30	1.20		NA		9	3b	10	3b		9	2	10	2	
4	7	0.10	0.20	12	0.05	0.20	10	3b	12	3b		NA		NA		
5	8	1.00	1.00		NA		9	3b	NA			9	2	NA		
6	8	1.30	1.40		NA		11	3b	NA			11	2	NA		
7	8	0.48	0.40	14	0.80	0.70	9	3a	14	3b	✓	9	1	14	1	
8	8	0.48	0.48	15	1.40	1.60	16	3a	NA			16	2	NA		
9	9	0.60	0.20	13	1.10	1.00	9	3b	12	3b		9	2	13	2	
10	9	0.70	0.70	13	1.10	1.20	9	3b	NA			9	2	13	2	
11	9	0.48	0.48	14	1.30	1.20	9	3b	13	3b		9	2	13	2	
12	9	1.00	1.00	14	1.10	0.90	10	3b	13	3b		10	2	12	2	
13	9	0.48	0.48	16	0.80	0.80	10	3a	14	3b	✓	10	1	16	1	
14	9	0.60	0.48	16	1.00	1.00	10	3b	NA			10	2	NA		
15	10	0.80	0.80		NA		10	3a	NA			10	1	NA		
16	10	1.00	1.00	11	1.00	1.10	11	3b	NA			11	2	NA		
17	10	0.30	0.30	14	0.30	0.30	10	3b	NA			10	2	NA		

18	11	0.50	0.40		NA		11	3b	NA			11	2	NA	
19	11	0.30	0.30		NA		11	3a	NA			NA		NA	
20	11	0.78	0.78		NA		11	3b	12	3b		11	2	12	2
21	11	0.54	0.12	13	0.80	0.30	13	2	NA			13	2	NA	
22	11	1.00	1.00	16	1.00	NA	13	3b	15	3b		15	2	NA	
23	12	0.18	0.18		NA		16	3b	NA			NA		NA	
24	12	1.00	0.90	15	1.00	1.00	12	3b	15	3b		12	2	15	2
25	12	1.30	1.30	16	1.30	1.18	12	3b	15	3b		13	2	16	2
26	12	1.00	1.00	16	1.00	1.00	12	3a	16	3b	✓	16	2	NA	
27	13	0.50	0.60		NA		13	3a	NA			13	1	NA	
28	13	1.30	1.10	15	1.10	0.88	14	3b	NA			14	2	15	2
29	13	1.00	1.18	16	0.78	0.78	15	3b	NA			NA		NA	
30	14	0.90	0.80		NA		15	3b	NA			15	2	NA	
31	14	0.48	0.48		NA		14	3a	NA			14	1	NA	
32	14	1.00	1.00	15	1.00	1.00	NA		NA			NA		NA	
33	14	0.18	0.20	16	0.36	0.36	16	3a	NA			16	1	NA	
34	15	1.00	1.00		NA		15	3b	NA			15	2	NA	
35	15	0.50	0.50		NA		15	3a	NA			15	1	NA	
36	15	1.00	1.00	16	1.00	1.00	16	3b	NA			16	2	NA	
37	15	0.18	0.18	16	0.75	0.56	15	3b	16	3b		16	1	NA	
38	15	1.00	1.00	16	1.08	1.00	16	3b	NA			NA		NA	
39	15	0.80	0.80	16	0.80	0.80	15	3b	NA			15	1	NA	
40	16	0.48	0.48		NA		NA		NA			NA		NA	

41	16	1.00	1.00	NA	NA		NA	NA		NA
42	16	0.18	0.18	NA	16	3a	NA	16	1	NA

AF type = autofluorescence type; BL = baseline; Fds type = fundus type; FU = follow-up; L = left; NA; LogMAR VA = logarithm of the minimum angle of resolution visual acuity; NA = not available; Pt = patient; R = right;
