# Supplementary material for: Clinical and Molecular Characteristics of Childhood-Onset Stargardt Disease
Source: Ophthalmology. Author manuscript; Available in PMC 2015 Jun 8. (PMC4459618; doi:10.1016/j.ophtha.2014.08.012)
Supplement: Table 4 [file NIHMS693250-supplement-Table_4.pdf]

Table 4. Suggested Pathogenicity of the 46 *ABCA4* Variants Identified in Childhood-onset Stargardt Disease

Exon/ IVS	Nucleotide substitution	Protein change/effect	Number of alleles identified	Pt	Reference	SIFT	Polyphen2		HSF			Allelic frequency observed by EVS	db SNP
						Prediction	Prediction	Hum var score (0-1)	Wild type CV	Mutant CV	effect		
1	c.21dupA	p.Gln8fs	1	20	This study							ND	
6	c.634C>T	p.Arg212Cys	1	7	Simonelli F et al.[30]	Not tolerated	PRD	0.951				0.0116	rs61750200
6	c.768G>T	Splice	1	17	Klevering et al.[28]				91.6	80.7	weakens the splice donor site by ~12%	ND	
10	c.1317G>A	p.Trp439*	1	21	Fujinami et al.[5]							ND	
11	c.1531C>T	p.Arg511Cys	1	37	Zernant et al.[22]	Not tolerated	PRD	0.976				ND	
12	c.1557C>A	p.Cys519*	1	12	This study							ND	
12	c.1648G>A	p.Gly550Arg	1	26	Shroyer et al.[27]	Not tolerated	POD	0.882	0	81.58	creates a new splice acceptor site	ND	
12	c.1757A>G	p.Asp586Gly	1	39	This study	Not tolerated	POD	0.599				ND	
12	c.1760G>A	p.Arg587Lys	1	29	This study	Not tolerated	POD	0.749	84.6	74	weakens the splice donor site by ~13%	ND	
13	c.1906C>T	p.Gln636*	3	2, 18, 30	Zernant et al.[22]							0.0116	rs145961131
14	c.1957C>T	p.Arg653Cys	1	36	Rivera et al.[25]	Not tolerated	PRD	0.999				ND	
16	c.2564G>A	p.Trp855*	1	29	Rivera et al.[25]								rs61752406
17	c.2588G>C	p.Gly863Ala/ p.Gly863del	2	25, 27	Lewis et al.[24]/Maugeri et al.[34]	Not tolerated	POD	0.864				0.6744	rs76157638
18	c.2712delG	p.Glu905fs	2	5	This study							ND	
19	c.2861A>C	p.Tyr954Ser	1	16	Aguirre-Lamban et al.[32]	Not tolerated	PRD	0.959				ND	
21	c.3056C>T	p.Thr1019Met	1	31	Rozet et al.[23]	Not tolerated	PRD	1.000				ND	rs201855602
21	c.3064G>A	p.Glu1022Lys	2	28	Webster et al.[26]	Not tolerated	PRD	1.000				ND	rs61749459

21	c.3081T>G	p.Tyr1027*	2	8	This study							ND	
22	c.3197T>G	p.Met1066Arg	1	38	This study	Not tolerated	POD	0.495				ND	
22	c.3259G>A	p.Glu1087Lys	1	10	Lewis *et al.* 1999	Not tolerated	PRD	0.997				ND	rs61751398
22	c.3289A>T	p.Arg1097*	1	6	This study							ND	
22	c.3322C>T	p.Arg1108Cys	2	11, 13	Rozet et al.[23]	Not tolerated	PRD	0.986				0.0116	rs61750120
23	c.3364G>A	p.Glu1122Lys	1	25	Lewis et al.[24]	Not tolerated	PRD	1.000				ND	rs61751399
23	c.3385C>T	p.Arg1129Cys	1	42	Zernant et al.[22]	Not tolerated	PRD	0.998				ND	
28	c.4139C>T	p.Pro1380Leu	3	21, 34, 36	Lewis et al.[24]	Not tolerated	PRD	0.99				0.0233	rs61750130
28	c.4216C>T	p.His1406Tyr	1	14	Lewis et al.[24]	Not tolerated	POD	0.824				ND	rs61750133
28	c.4222T>C	p.Trp1408Arg	1	14	Lewis et al.[24]	Not tolerated	PRD	0.973				ND	rs61750135
30	c.4363T>C	p.Cys1455Arg	2	17, 24	Fujinami et al.[5]	Not tolerated	PRD	0.999				ND	
30	c.4469G>A	p.Cys1490Tyr	4	9, 11, 38, 42	Lewis et al.[24]	Not tolerated	PRD	0.994				ND	rs61751402
31	c.4577C>T	p.Thr1526Met	1	13	Lewis et al.[24]	Not tolerated	PRD	0.999				ND	rs61750152
36	c.4918C>T	p.Arg1640Trp	1	14	Briggs et al.[22]	Not tolerated	PRD	0.999				ND	
36	c.5160_5161delCA	p.Thr1721fs	1	27	This study							ND	rs61750566
37	c.5308T>G	p.Tyr1770Asp	1	34	This study	Not tolerated	PRD	1.000				ND	
37	c.5213_5214insTGC	p.Ala1739dup	1	37	This study							ND	
42	c.5882G>A	p.Gly1961Glu	5	31, 33, 35, 37, 39	Lewis et al.[24]	Not tolerated	PRD	1.000				0.4186	rs1800553
44	c.6079C>T	p.Leu2027Phe	3	22, 40, 41	Lewis et al.[24]	Not tolerated	PRD	1.000				0.0349	rs61751408
44	c.6088C>T	p.Arg2030*	2	2, 25	Lewis et al.[24]							ND	rs61751383
45	c.6215G>A	p.Ser2072Asn	1	32	This study	Not tolerated	PRD	1.000				ND	
47	c.6449G>A	p.Cys2150Tyr	1	26	Fishman et al.[16]	Not tolerated	PRD	1.000				0.0116	rs61751384
IVS35	c.5018+2T>C	splice	1	32	Fujinami et al.[8]				81.15	0	eliminates the splice donor site	ND	

IVS36	c.5196+1G>A	splice	1	6	Shroyer et al.[27]				83.28	0	eliminates the splice donor site	ND	
IVS38	c.5461-10T>C	Uncertain	9	7, 9, 10, 15, 16, 18, 20, 22, 41	Briggs et al.[20]							0.0349	rs1800728
IVS40	c.5714+5G>A	splice	1	24	Cremers et al.[19]				85.49	73.33	weakens the splice donor site by ~14%	0.1512	
IVS47	c.6479+1G>A	splice	2	3	Zernant et al.[22]				87.25	0	eliminates the splice donor site	ND	
IVS48	c.6729+4_6729+18delAGTTGGCCCTGGGGC	splice	1	33	Littink et al.[31]							ND	
IVS49	c.6817-2A>C	splice	1	30	This study				93.6	0	eliminates the splice acceptor site	ND	

CV = consensus value; EVS = Exon variant server; Het = heterozygous; Hom = homozygous; HSF = human splicing finder; Hum Var Score = human var score; IVS = intervening sequence; NA = not applicable; ND= not detected; POD = possibly damaging; PRD = probably damaging; Pt = patient; SIFT = Sorting Intolerant From Tolerant ; WT = wild type.

SIFT (version 4.0.4) results are reported to be tolerant if tolerance index ≥ 0.05 or intolerant if tolerance index < 0.05. [http://sift.bii.a-star.edu.sg/www/SIFT_BLink_submit.html/. Accessed February 1, 2013.] Polyphen 2 (vision 2.1) appraises mutations qualitatively as Benign, Possibly Damaging or Probably Damaging based on the model's false positive rate. [http://genetics.bwh.harvard.edu/pph2/. Accessed November 1, 2013.] HumanVar-trained model of Polyphen 2 was selected, since diagnostics of mendelian diseases requires distinguishing mutations with drastic effects from all the remaining human variation, including abundant mildly deleterious alleles. The cDNA is numbered according to Ensemble transcript ID ENST00000370225, in which +1 is the A of the translation start codon. Human Splicing Finder (HSF, version 2.4.1) reports the results from the HSF matrix: the higher the consensus value, the stronger the predicted splice site. The values for the wildtype and mutant sequences are shown; the larger the difference between these values, the greater the chance that the variant can affect splicing [http://www.umd.be/HSF/. Accessed November 1, 2013.]. EVS denotes the allele frequencies of variants on the Exome Variant Server, NHLBI Exome Sequencing Project, Seattle, WA, USA. [http://snp.gs.washington.edu/EVS/. Accessed Febrary 1, 2013.]
