# Supplementary material for: Clinical and Molecular Characteristics of Childhood-Onset Stargardt Disease
Source: Ophthalmology. Author manuscript; Available in PMC 2015 Jun 8. (PMC4459618; doi:10.1016/j.ophtha.2014.08.012)
Supplement: Table 5 [file NIHMS693250-supplement-Table_5.pdf]

Table 5. Comparison of Clinical Characteristics, Genotype, and Allele Frequency of the Prevalent Variants between Childhood-onset and Adult-onset Stargardt Disease with two or more disease-causing *ABCA4* variants.

	Childhood-onset Stargardt disease (n=34)						Adult-onset Stargardt disease (n=64)					
Median age of onset (yrs)	8.5 (3-14)						27.0 (17-65)					
Median age at examination (yrs)	11.5 (7-16)						44.0 (21-71)					
Median LogMAR VA of the right eye	0.79 (0.18-1.30)						1.00 (-0.08-2.00)					
Fundus Appearance	Total (n=32)						Total (n=64)					
	Grade 1	Grade 2	Grade 3a	Grade 3b	Grade 3c	Grade 4	Grade 1	Grade 2	Grade 3a	Grade 3b	Grade 3c	Grade 4
	1	1	8	22	0	0	0	6	4	34	12	8
	Flecks						Flecks					
	Macular 2		Peripheral 21		No flecks 9		Macular 14		Peripheral 39		No flecks 11	
	Pigmentation 2				No pigmentation 30		Pigmentation 29				No pigmentation 35	
Autofluorescence Pattern	Total (n=29)						Total (n=62)					
	Type 1		Type 2		Type 3		Type 1		Type 2		Type 3	
	8		21		0		20		33		9	
OCT, CFT (μm) of the right eye	Total (n=19)						Total (n=33)					
	61.0 (33-138)						81.0 (20-297)					
ERG group	Total (n=18)						Total (n=59)					
	Group 1		Group 2		Group 3		Group 1		Group 2		Group 3	
	7		1		10		34		7		18	
Genotype group classification	Total (n=34)						Total (n=64)					
	Group A		Group B		Group C		Group A		Group B		Group C	
	7		15		12		3		26		35	
Frequencies of the most prevalent variants	c.5461-10T>C		p.Gly1961Glu		p.Cys1490Tyr		p.Gly1961Glu		p.Gly863Ala		p.Leu2027Phe	
	8 (11.8%)		4 (5.9%)		4 (5.9%)		16 (12.5%)		13 (10.1%)		8 (6.3%)	

AF type = autofluorescence type; CFT = central foveal thickness; ERG = electroretinogram; LogMAR VA = logarithm of the minimum angle of resolution visual acuity; OCT = optical coherence tomography.

In order to investigate the differences between the patients with childhood-onset Stargardt Disease (STGD) and those with adult-onset STGD, clinical and molecular genetic data of patients with adult-onset STGD ascertained at Moorfields Eye Hospital were reviewed. The comparison group consisted of all patients who had adult-onset STGD (older than 17 years old), and two or more disease-causing *ABCA4* variants. For the purpose of this comparison, 34 patients with childhood-onset STGD and two or more disease-causing *ABCA4* variants were selected.
